# Innate immune responses to paraquat exposure in a *Drosophila* model of Parkinson’s disease

**DOI:** 10.1038/s41598-019-48977-6

**Published:** 2019-09-03

**Authors:** Urmila Maitra, Michael N. Scaglione, Stanislava Chtarbanova, Janis M. O’Donnell

**Affiliations:** Department of Biological Sciences, University of Alabama, Tuscaloosa, Alabama 35487-0344 United States

**Keywords:** Neuroscience, Parkinson's disease

## Abstract

Parkinson’s disease (PD) is a progressive, neurodegenerative movement disorder characterized by the loss of dopaminergic (DA) neurons. Limited understanding of the early molecular pathways associated with the demise of DA neurons, including those of inflammatory exacerbation of neurodegeneration, is a major impediment to therapeutic development. Recent studies have implicated gene-environment interactions in PD susceptibility. We used transcriptomic profiling in a *Drosophila* PD model in response to paraquat (PQ)-induced oxidative stress to identify pre-symptomatic signatures of impending neuron dysfunction. Our RNAseq data analysis revealed extensive regulation of innate immune response genes following PQ ingestion. We found that PQ exposure leads to the activation of the NF-κB transcription factor, Relish, and the stress signaling factor JNK, encoded by the gene *basket* in *Drosophila*. Relish knockdown in the dopaminergic neurons confers PQ resistance and rescues mobility defects and DA neuron loss. Furthermore, PQ-induced toxicity is mediated through the immune deficiency signaling pathway. Surprisingly, the expression of Relish-dependent anti-microbial peptide (AMPs) genes is suppressed upon PQ exposure causing increased sensitivity to Gram-negative bacterial infection. This work provides a novel link between PQ exposure and innate immune system modulation underlying environmental toxin-induced neurodegeneration, thereby underscoring the role of the innate immune system in PD pathogenesis.

## Introduction

Parkinson’s disease (PD) is an age-associated neurodegenerative disorder primarily caused by the progressive loss of dopaminergic (DA) neurons from the *substantia nigra* in the brain^1^. The clinical motor symptoms include rest tremor, rigidity, progressive bradykinesia and postural instability. Apart from the motor deficits, PD is also linked to several non-motor symptoms, including sleep disorder, depression, constipation, anxiety, impaired reaction time, that often manifest during the early pre-clinical stages of PD^2^. Although the precise molecular mechanisms involved in the neurodegenerative process remain unclear, there is increasing evidence that PD is a complex multifactorial disorder caused by a combination of genetic and environmental factors, which affect multiple key signaling pathways in different cell types leading to the loss of DA neurons^3,4^. One of the major challenges to therapeutic development is the lack of clear understanding of the pre-symptomatic molecular pathways that are either triggered or suppressed in PD pathogenesis.

Chronic neuroinflammation is a common emerging hallmark of several neurodegenerative diseases, including PD^5^. Post-mortem studies of brains indicate activation of innate immune glial cells and elevated levels of pro-inflammatory factors as common features of PD patients^6,7^. Activated glial cells induce inflammatory responses to promote defense against pathogen invasion or tissue damage^8^. However, improper resolution leads to uncontrolled persistent inflammation that contributes to neurotoxicity. Expression of several Toll-like receptors (TLRs), which are responsible for initiating the inflammatory response, has been observed in post-mortem PD brain samples^9^. Moreover, TLR4 has been implicated in the abnormal deposition of α-synuclein, a protein that accumulates in brain cells of PD patients^10^. Consistent with these findings, several recent studies demonstrate that anti-inflammatory compounds exhibit significant protective functions for DA neurons in PD models. Treatment with the synthetic anti-inflammatory steroid, dexamethasone, had a beneficial effect against neurodegeneration and reduced activation of glial cells in mouse PD models^11^. Administration of minocycline, a tetracycline derivative, effectively protects DA neurons in both mouse and *Drosophila* models by regulating the mitogen-activated protein kinase (MAPK) signaling pathways, which plays a critical role in controlling the expression of pro-inflammatory genes^12,13^. In mammalian PD models, activation of c-Jun N-terminal Kinase (JNK) has been implicated in PQ-induced oxidative stress and neurodegeneration^14^. Moreover, in transgenic mice models, activated JNK has been detected in a leucine-rich repeat kinase 2 (LRRK2) mutant, a gene linked to autosomal dominant familial PD^15^. These findings provide strong link between dysregulated inflammatory responses and PD pathogenesis.

Over the last 15 years, investigations of neurodegenerative disease have incorporated invertebrate models such as *Drosophila melanogaster* and *Caenorhabditis elegans* on the principle that cellular and molecular mechanisms of neurodegeneration, metabolism, stress response and neuronal function are highly conserved^16–18^. These models offer powerful genetic tools and short generation times to provide an entrée into genetic screens for identifying potentially important network components and cellular responses that may then be validated in cell culture or *in vivo* mammalian models. Significant effort has been devoted to identifying early signatures of neurodegenerative disease onset, with the goal of intervention at a point presumably more amenable to modulation or cure. RNA sequencing (RNAseq) transcriptome analysis has emerged as a powerful strategy to investigate differential gene regulation and to identify early and predictive molecular signatures of neurodegenerative disease^19,20^. However, most of the reported transcriptomic studies have mainly focused on genetic PD models, and it is not clear whether genetic mutation and environmental insult individually trigger the same molecular responses or distinct, interacting pathways.

There is accumulating evidence for increased innate immune activation in PD^21^. In *Drosophila*, the innate immune response to bacterial and fungal pathogens is mainly controlled by two signaling cascades, the Toll and the immune deficiency (IMD) pathways, both of which activate members of the Nuclear Factor kappa B (NF-κB) family of transcription factors^22^. The Toll pathway primarily responds to infections with fungi and Gram-positive bacteria, while the IMD pathway is predominantly activated by infection with Gram-negative bacteria. Peptidoglycans derived from the Gram-negative bacterial cell wall bind to the transmembrane peptidoglycan recognition protein (PGRP)-LC or the intracellular PGRP-LE receptors. Following receptor dimerization, the adaptor protein IMD initiates intracellular signaling by associating with Fas-associated death domain protein (FADD), which then recruits the caspase-8 homolog death related ced-3/Nedd2-like caspase (DREDD). DREDD cleaves off the amino-terminal portion of IMD and stabilizes through IMD ubiquitination. Activated DREDD also cleaves the inhibitory C-terminal domain of Relish, a homologue of mammalian NF-κB^23^. Relish, the key transcription factor activated by stimulation of the IMD signaling pathway, undergoes nuclear translocation upon activation, leading to the transcription and synthesis of antimicrobial peptides (AMPs)^24^. In mammalian models, NF-κB also plays an essential role in the inflammatory response through regulation of genes encoding pro-inflammatory mediators^25^. Increased NF-κB activation has been detected in the brains of PD patients, emphasizing the link between immune activation and neurodegenerative disease^26^. Similarly, increased NF-κB activation has been linked to age-related neurodegeneration in *Drosophila*^27^. Therefore, tight regulation of the IMD pathway is crucial to avoid uncontrolled chronic inflammation. The immune pathways are regulated by several negative feedback loops to avoid prolonged inflammatory responses^28^. Earlier studies have shown that flies with mutations in a gene that encodes a repressor of the IMD pathway, *defense repressor* 1 (*dnr1*) display neurodegeneration due to increased expression of Relish target genes encoding AMPs^29^. In addition, flies with mutations in *ATM*, a gene associated with the neurodegenerative disease ataxia-telangiectasia (A-T) in humans, show elevated levels of AMPs in glial cells demonstrating that Relish is involved in the development of the degenerative disease pathology^30,31^. A recent study in humans links the immune system to PD by demonstrating a heightened immune response to α-synuclein^32^. We have previously reported that PQ treatment induces an inflammatory response that parallels mammalian neuroinflammation through the induction of nitric oxide synthase (NOS), a critical component of the *Drosophila* innate immune response^33^. Increasing evidence suggests a critical role of chronic intestinal inflammation to PD pathogenesis^34^. It has also been hypothesized that activation of innate immune genes could play a neuroprotective role^35^. Altogether, these findings demonstrate that dysregulation of immune pathways in *Drosophila* also can contribute to neurodegeneration, as in mammals. Given the striking parallels of *Drosophila* and mammalian inflammatory responses during neurodegeneration, we postulated that controlled induction of neurodegeneration in *Drosophila* would be an entrée into the earliest cellular responses to neuron insult and that many of these responses will be conserved.

Epidemiological studies have identified the herbicide, paraquat (PQ), as a potential environmental risk factor in the onset of PD^36–39^. Previously, we found that exposure of adult flies to low doses of PQ (3–20 mM) resulted in a recapitulation of parkinsonism movement disorders, including tremors, loss of balance and slowed locomotion, accompanied by loss of dopaminergic neurons, with regionally specific differences in neuron susceptibility^3,13^. In the present study, we employed transcriptomic profiling of gene expression responses to transient PQ exposure using *Drosophila* heads to build a temporal landscape of pre-symptomatic gene responses to neuron damage – that is, prior to acquisition of movement abnormalities. Our RNAseq data analysis revealed a predominant genetic signature of innate immune response genes immediately following PQ exposure. Interestingly, we found that PQ treatment leads to Relish and JNK activation, and that targeted knockdown of *relish* in DA neurons confers increased resistance to PQ. Moreover, PQ-induced toxicity involves components of the IMD signaling pathway. Strikingly, activated Relish fails to induce AMP expression leading to increased sensitivity to Gram-negative bacterial infection following PQ treatment. Together, our findings indicate a novel link between environmental-toxin induced PD pathogenesis and innate immune gene regulation, leading to increased susceptibility to bacterial infection.

## Results

### Transcriptomic profiling reveals early gene expression responses to transient paraquat exposure

**Figure 1:**
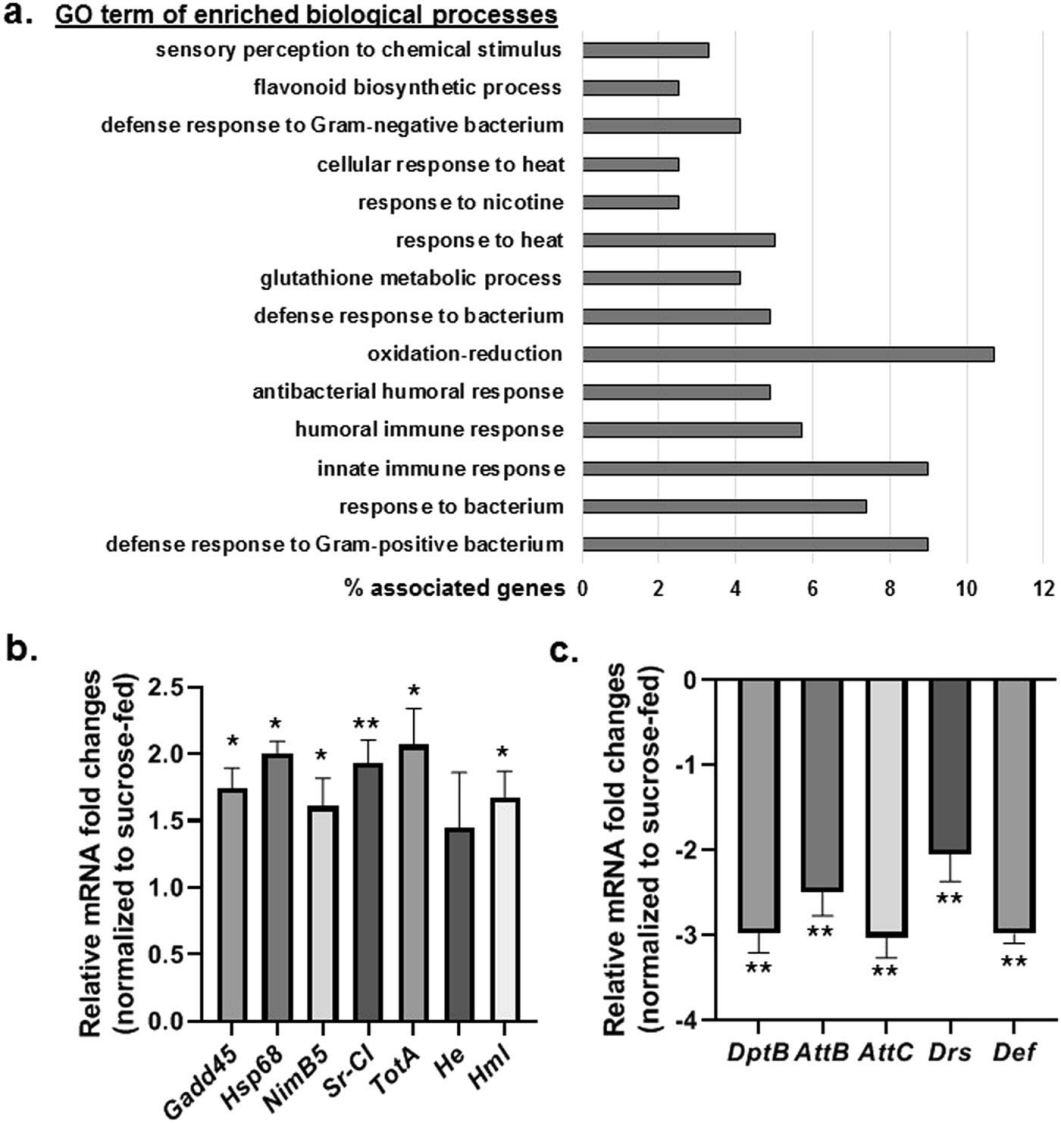
Paraquat exposure regulates innate immune genes in a *Drosophila* model of PD. **(a)** Gene ontology (GO) biological processes enrichment analysis of PQ exposure in adult male *Drosophila* heads show extensive regulation of innate immune response genes. RNAseq analysis were performed using four independent biological samples from both sucrose (control) and PQ-fed flies. GO analysis was performed on the differentially expressed mRNAs between sucrose (control) and PQ-fed samples using DAVID v6.8 functional annotation analysis. Bars represent the percentage of associated genes for enriched biological processes in the PQ-fed group. **(b**,**c)** Validation of selected RNAseq-based candidate gene expression that are either upregulated or downregulated by qRT-PCR. RNA was isolated from heads of *Canton S* WT male flies following treatment with 2.5% sucrose (control) or 5 mM PQ in 2.5% sucrose for 12 h. Selected differentially expressed transcripts identified through RNAseq profiling were verified using qRT-PCR and plotted after normalization with *rp49* levels as the internal control. Each data point represents the mean +/− SEM. Values represent the fold-change of indicated genes after PQ exposure compared with sucrose-fed controls. mRNA fold changes are normalized to the sucrose-fed flies (assigned a value of 1). Statistical significance between the sucrose and PQ-fed groups were calculated using the Mann-Whitney U test. *P < 0.05; **P < 0.01. The results are the averages of at least three independent biological replicates.

In order to capture the early, pre-degenerative phase of differential gene responses to PQ exposure, we performed RNAseq profiling of heads from the wild type (WT) *Drosophila* strain, *Canton S*, that were fed either 2.5% sucrose (control) or 5 mM PQ in 2.5% sucrose for 12 h (Fig. S1). We used male flies in our study because we previously determined that they are more sensitive to PQ toxicity during the first week of adult life and display parkinsonian symptoms earlier than female flies^3^. Gene ontology (GO) enrichment analysis was performed on the list of differentially expressed mRNAs using Database for Annotation, Visualization and Integrated Discovery (DAVID) v6.8 (Fig. 1a). We identified 124 genes that were differentially regulated in response to PQ (Supplemental Table 1); these were enriched for biological processes (BP) that include innate immunity, oxidation-reduction, glutathione metabolic process, response to heat and response to nicotine based on Gene ontology (GO) analysis (Fig. 1a and Table 1). The GO terms represented in Table 1 were filtered with cut-off thresholds that only show BP categories with 3 or more associated genes. Using quantitative real time polymerase chain reaction (qRT-PCR), we subsequently validated PQ-responding candidate genes that were either upregulated or downregulated for the following enriched BP categories, including, the innate immune response and antibacterial defense response (*nimrodB5*, *sr-CI*, *totA*, *hemolectin*, *diptericinB*, *attacin-B*, *attacin-C*, *drosomycin*, *defensin*), oxidation-reduction (*Gadd45*) and response to heat (*Hsp68*) (Fig. 1b,c).

**Table 1:** Differentially regulated genes in response to PQ treatment.

GO term of enriched biological processes	Associated Genes
defense response to Gram-positive bacterium	**Up**: *TotM*, *CG30098* **Down**: *AttA*, *AttB*, *AttC*, *AttD*, *CG6639*, *CecA1*, *Def*, *DptB*, *CG32185*
response to bacterium	**Up**: *TotA*, *TotM*, *TotX*, *Sr-CI*, *CG11501* **Down**: *AttC*, *CecA1*, *Def*, *Drs*
innate immune response	**Up**: *He*, *TotM*, *TotA*, *TotX* **Down**: *AttA*, *AttB*, *AttC*, *PGRP-SC2*, *CecA1*, *Def*, *Drs*
humoral immune response	**Down**: *AttA*, *AttB*, *AttC*, *Def*, *DptB*, *CecA1*, *CG32185*
antibacterial humoral response	**Down**: *AttA*, *AttB*, *AttC*, *Def*, *Drs*, *CecA1*
oxidation-reduction	**Up**: *CG10337*, *CG12766*, *CG15343*, *CG3397*, *Cyp12c1*, *Cyp6d2*, *Cyp309a1*, *Cyp4e3*, *Cyp4g1*, *Cyp9b2*, *ImpL3*, *Gadd45*, *Nmdmc*
defense response to bacterium	**Up**: *TotA*, *NimB5* **Down**: *AttA*, *AttB*, *CecA1*, *Def*
glutathione metabolic process	**Up**: *GstD2*, *GstD5*, *GstE7*, *GstE8* **Down**: *GstE4*
response to heat	**Up**: *TotM*, *TotA*, *TotX*, *Hsp68*, *Hsp22*, *Hsp26*
response to nicotine	**Up**: *NtR*, *nAChRbeta3* **Down**: *CG8147*
cellular response to heat	**Up**: *TotA*, *TotX*, *TotM*
defense response to Gram-negative bacterium	**Up**: *Sr-CI* **Down**: *AttA*, *Drs*, *CecA1*, *CG32185*
flavonoid biosynthetic process	**Up**: *CG5999*, *Ugt86Dd*, *Ugt37b1*
sensory perception of chemical stimulus	**Down**: *Obp28a*, *Obp69a*, *Obp83a*, *Obp83b*

Among the induced expression of genes appearing in the GO terms of enriched BP of oxidation-reduction was the Growth Arrest and DNA-Damage-inducible protein 45 (*Gadd45)* transcript (Table 1 and Fig. 1b). Mammalian GADD45 functions in DNA-repair in response to environmental and physiological stress, and overexpression of its *Drosophila* ortholog in the nervous system has been shown to extend life span, delay age dependent neurodegeneration and increase resistance to oxidative stress^40–42^. A targeted knockdown of *Gadd45* in dopaminergic neurons revealed decreased survival following PQ exposure (Fig. S2), suggesting a neuroprotective effect of GADD45 against PQ induced neurotoxicity, in parallel with prior reports of *Drosophila* GADD45 function in the nervous system. Consistent with our findings, GADD45α has also been shown to protect M17 human dopamine neuroblastoma cells against 1-methyl-4-phenylpyridinium (MPP+) toxicity^43^.

We also identified genes encoding three heat shock proteins (*Hsp* *22, 26, and 68*), and three other turandot genes (totA, tot M, and totX) in the response to heat category, that were induced in response to PQ exposure (Table 1). There is increasing evidence that Hsps play protective roles in response to neurodegenerative diseases including PD, due to their function as molecular chaperones regulating proper protein folding^44^. Neuron-specific expression of Hsp26 increases life span and enhances stress resistance in *Drosophila*^45^. In addition, Hsp70 has been shown to be protective against both PQ-induced neurodegeneration in *Drosophila* and α-synuclein-induced cellular toxicity in mammalian models^46,47^.

Another subset of five genes (*glutathione-S-transferase D2*, *D5*, *E4*, *E7* and *E8*) that were differentially regulated by PQ belong to the BP category of glutathione metabolic process. Glutathione *S*-Transferases (GSTs) are a family of enzymes that catalyze the addition of glutathione to a variety of cellular substrates, including products of reactive oxygen species^48^. Consistent with our findings, increase in GST activities have been reported in the brains of human PD patients, possibly due to increased levels of oxidative stress in PD pathogenesis^48,49^.

Another candidate gene, *metallothionein D (mtnD)*, that was not included in Table 1 since it was eliminated due to the three or more associated gene number cut-off threshold for BP categories, is worth noting, as it was strongly induced (9.5 fold compared to sucrose-fed) by PQ ingestion. Metallothioneins are metal-binding proteins shown to be associated with neuroprotection and increased expression of *mtnD* has been reported in the substantia nigra and cortex of sporadic PD cases^50,51^. These findings underscore the similarities between the mammalian and the *Drosophila* disease model due to highly conserved gene responses linked to PD pathogenesis.

The GO term enrichment analysis of biological processes also revealed extensive regulation of innate immune response genes following PQ feeding, with a total of 17 genes that were either induced or repressed (Fig. 1a and Table 1). Many of these genes are expressed predominantly in *Drosophila* hemocytes, which are functional analogs of mammalian macrophages that show inflammatory responses to PQ exposure. Induced genes expressed in the hemocytes included *hemese* (*he*), *hemolectin* (*hml)*, two *nimrod* genes, (*nimB5* and *nimC4*), three *turandot genes* (*totA*, *tot M*, and *totX*), and *scavenger receptor C1* (*sr-C1*), suggesting involvement of the *Drosophila* immune response in PQ-induced neurodegeneration (Fig. 1b and Table 1). The Nimrod and Turandot gene families are important components of the *Drosophila* innate immune response that participate in defense mechanisms through phagocytosis of either bacteria, apoptotic cells or both^52–55^.

Surprisingly, the RNAseq screen also revealed PQ-mediated down-regulation of multiple anti-microbial peptide (AMPs) genes downstream of both the IMD and the Toll innate immune pathways (Supplemental Table 1 and Fig. 1c). As part of innate immunity, AMPs are defensive peptides that are induced during inflammation or microbial challenge^24,56^. This result suggests that PQ-mediated suppression of AMPs might impair the host defense system as part of the aberrant neuroinflammatory responses. Taken together, these findings parallel growing evidence of the contribution of the dysregulated immune system in PD pathogenesis.

### Paraquat exposure results in activation of Relish/NF-kB and basket/JNK

**Figure 2:**
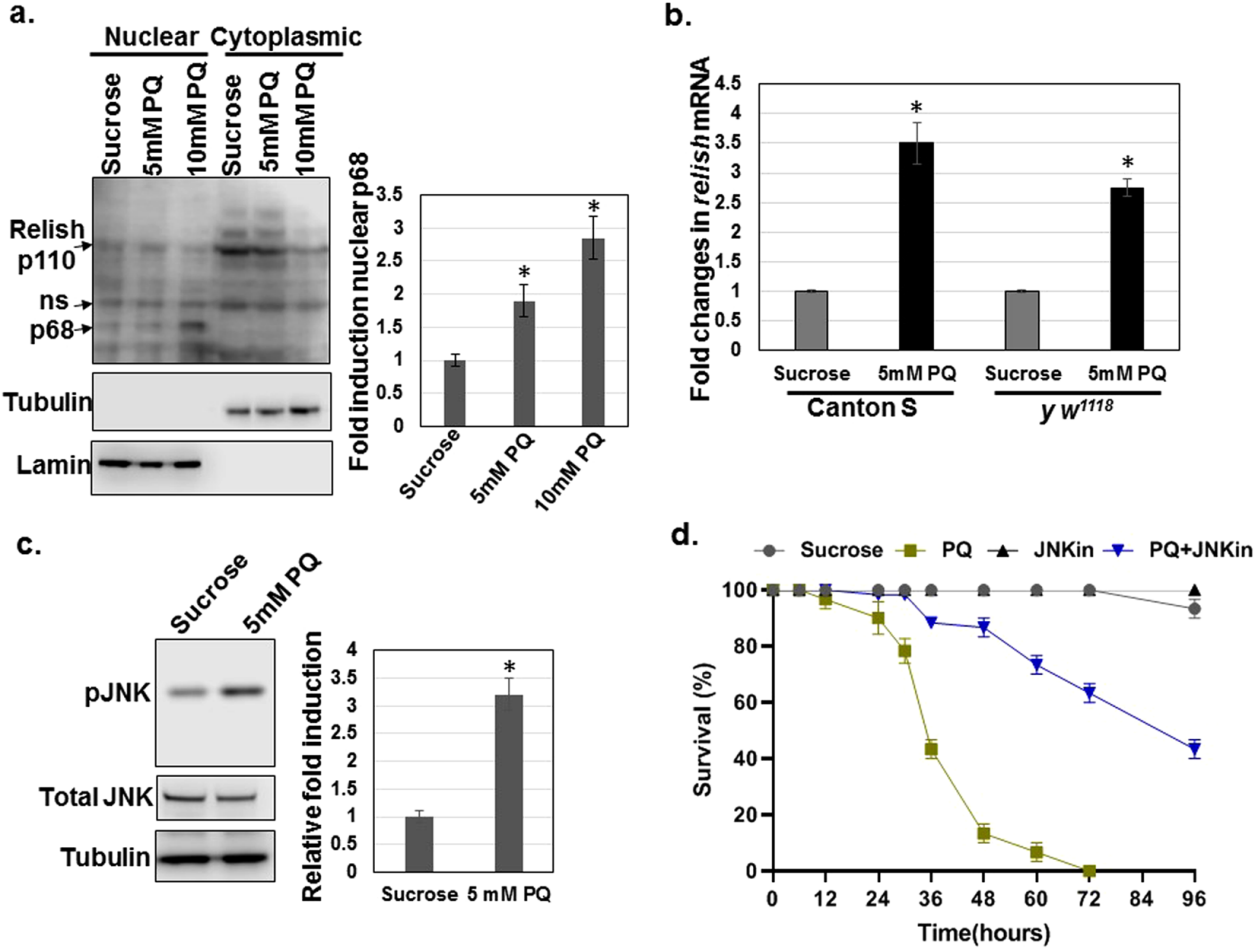
Paraquat activates immune signaling pathways. (**a)** Relish activation and nuclear translocation in response PQ treatment. Nuclear and cytoplasmic fractionation of heads from *Canton S* WT flies after sucrose or PQ (5 mM and 10 mM) ingestion for 12 h followed by immunoblot analysis using anti-Relish antibodies. The same blots were probed with anti-Lamin and anti-Tubulin as the nuclear and cytoplasmic markers, respectively for the fractionation protocol. The full-length Relish p110 and p68 are indicated by arrows and ns refers to non-specific band. Band intensities of nuclear Relish p68 were quantitated using the LI-COR C-DiGit Blot scanner software and plotted after normalization with corresponding Lamin band intensities. *p < 0.05 based on Student’s *t* test. Data are representative of three independent experiments. **(b)** PQ induces the expression of *relish* transcript. RNA was isolated from the heads of *Canton S* and *y w*^*1118*^ wild type flies following 2.5% sucrose or 5 mM PQ treatment for 12 h. The transcript levels of *relish* were analyzed using qRT-PCR and plotted after normalization with *rp49* levels as the internal control. Each data point represents the mean +/− SEM. mRNA fold changes are normalized to the sucrose-fed flies (assigned a value of 1) for each of the wild type strains. *P < 0.05 between the sucrose and PQ-fed group based on Mann-Whitney U test. **(c)** Paraquat induces JNK activation. Whole cell lysates were prepared using the heads of *Canton S* flies after 2.5% sucrose or 5 mM PQ ingestion for 12 h followed by immunoblot analysis using phospho-JNK antibodies. The same blots were probed with total JNK and Tubulin antibodies as the loading control. Band intensities were quantitated using the LI-COR C-DiGit Blot scanner software and plotted after normalization with total JNK band intensities. *P < 0.05 based on Student’s *t* test. Data are representative of three independent experiments. **(d)** Inhibition of JNK confers PQ resistance. *Canton S* flies were fed either 2.5% sucrose, 10 mM PQ, 10 μM JNK inhibitor (SP600125) or cofed PQ with the JNK inhibitor diluted in 2.5% sucrose, and survival was scored at the specified time points (hours). Log-rank test was used for survival analysis and statistically significant differences (*P* < 0.001) were observed between the PQ and PQ + JNKin group. Data are representative of at least five independent biological replicates.

Based on the transcriptomic analysis data that revealed differential regulation of immune system genes, with an upregulation of cellular immunity genes but a suppression of AMPs, we next explored the effect of PQ exposure on the levels of the transcription factor Relish, the fly ortholog of mammalian NF-κB. In *Drosophila*, Relish/NF-κB is mainly activated via the IMD pathway, where it plays a key role in regulating the humoral immune response through the production of AMPs, such as *attacin*, *diptericin*, and *cecropin*. Relish regulates the inducible immune response in *Drosophila* through signal-dependent translocation into the nucleus; its functions are highly conserved with mammalian NF-κB^56^. The full-length, 110 kD Relish protein is activated in the cytoplasm by proteolytic cleavage resulting in a 68 kD protein, which is subsequently translocated to the nucleus leading to the regulation of gene expression, and a 49 kDa protein, which remains cytosolic. To determine whether Relish is activated and translocated to the nucleus upon PQ treatment, we performed cellular fractionation using heads of *Canton S* WT male flies treated with either sucrose (control) or PQ (5 and 10 mM). Nuclear fractionation and immunoblotting data revealed that PQ exposure resulted in a dose-dependent induction of the active nuclear form, p68, while the cytoplasmic full-length p110 form decreased, confirming that PQ elicits activation and nuclear translocation of Relish (Fig. 2a). In addition, *relish* transcript levels were also induced (around 3-fold) in response to 5 mM PQ treatment in two different wild type strains, *Canton S* and *y w*^1118^ as shown in Fig. 2b. Since 5 mM PQ was capable of inducing Relish activation, we used this concentration for subsequent experiments to better distinguish early responses to PQ exposure. Although *relish* transcripts were not differentially regulated in our single time point RNAseq screen after normalization, we did observe a marginal induction in the raw RPKM values in the PQ-fed group compared to the sucrose-fed flies.

Activation of the *Drosophila* IMD pathway also triggers an inflammatory stress response via Jun N-terminal kinase (JNK), encoded by the gene *basket*, as the signaling pathway bifurcates from the IMD signaling cascade at the level of transforming growth factor β-activating kinase 1 (Tak1)^57^. We therefore investigated whether JNK signaling is affected since PQ exposure is known to induce oxidative stress. Our immunoblotting results show that JNK is activated through phosphorylation in response to PQ feeding as compared to sucrose-fed control flies (Fig. 2c). The same blots were probed with antibodies against total JNK and Tubulin as the loading control. In mammalian PD models, JNK signaling pathway has been shown to play an important role in regulating the cellular processes like oxidative stress and apoptosis^14^. However, JNK has been shown to be either protective or detrimental in diverse PD models, probably due to differences in cell type specificity, type of stimulus, duration of treatments and genetic backgrounds^13,14,47,58^. In order to decipher the role of JNK in PQ-mediated toxicity, *Canton S* WT flies were fed the JNK inhibitor (SP600125) alone or together with PQ and the survival was monitored. Consistent with earlier studies^59^, flies gained resistance to PQ in the presence of the JNK inhibitor, SP600125 (Fig. 2d). These data indicate that PQ exposure leads to activation of Relish and the stress response JNK pathway and that pharmacological inhibition of JNK enhances survival against PQ toxicity.

### Knockdown of *relish* in dopaminergic neurons confers paraquat resistance

**Figure 3:**
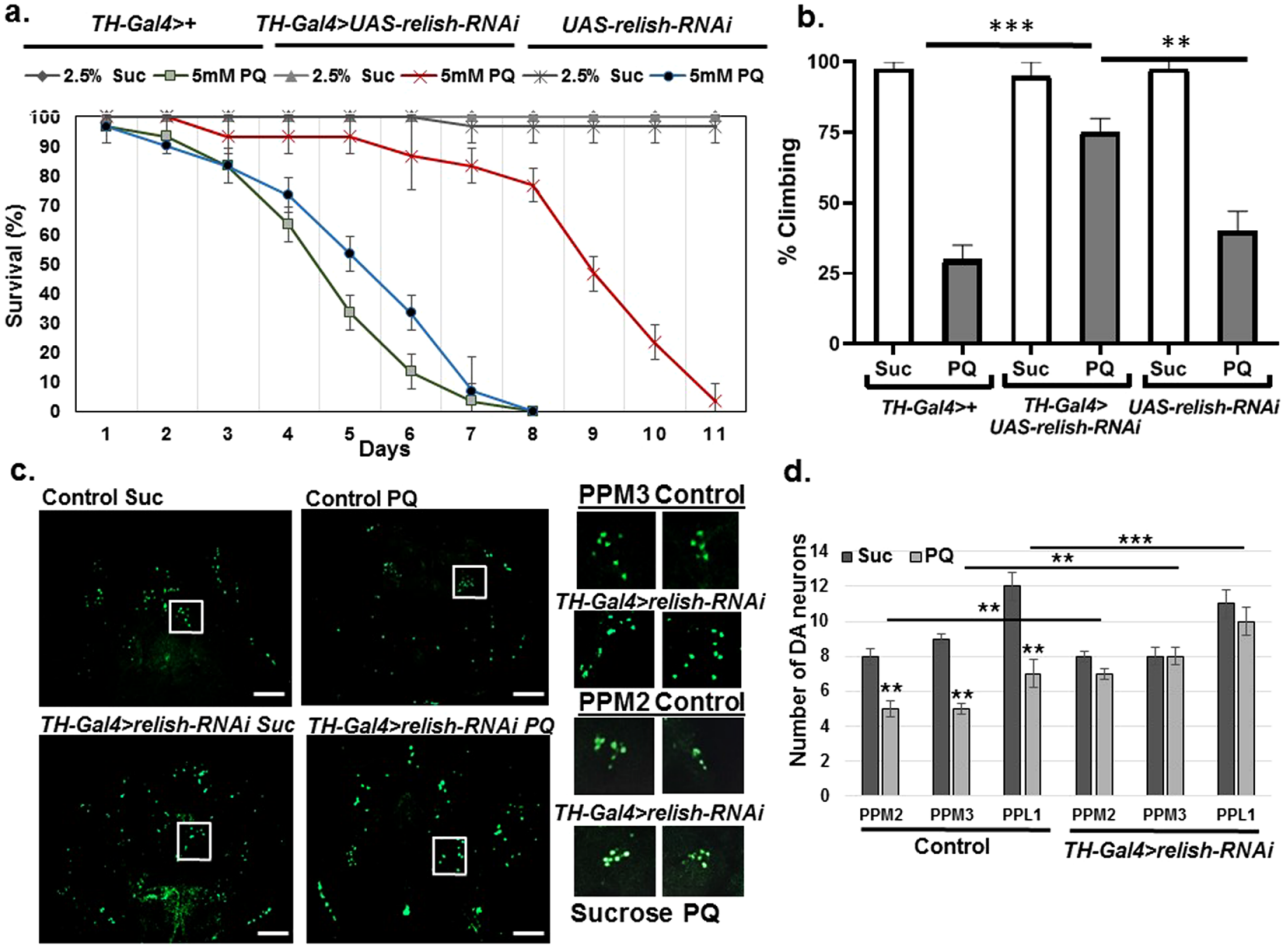
Targeted *relish* knockdown is protective against paraquat-mediated neurotoxicity. (**a)** Knockdown of *relish* in the DA neurons increases survival following PQ exposure. Survival assays were set up using male flies with *relish* knockdown in DA neurons (*TH-Gal4* > *UAS-relish-RNAi*) and the corresponding controls (*TH-Gal4*>+ and *UAS-relish-RNAi*) following sucrose or 5 mM PQ treatment. Log-rank test was used for survival analysis and statistically significant differences (*P* < 0.001) were observed between the *relish* knockdown (*TH-Gal4* > *UAS-relish-RNAi*) and the corresponding control groups (*TH-Gal4*>+ and *UAS-relish-RNAi*) in response to PQ exposure. Data are representative of at least five independent experiments with 10 flies per group. **(b)** Relish is involved in PQ-induced mobility defects. The effect of PQ on the mobility of controls (*TH-Gal4*/+ and *UAS-relish-RNAi*) and *relish* knockdown (*TH-Gal4 > UAS-relish-RNAi*) flies was determined by negative geotaxis assays. The number of flies able to climb 5 cm within 20 s was recorded using 10 flies per group. Data are representative of at least five independent experiments with 10 male flies per group. **P < 0.01; ***P < 0.001 based on one-way ANOVA between indicated genotypes in response to PQ. **(c)** Relish is associated with PQ-induced loss of DA neurons. The effect of PQ on DA neuron clusters in controls (*TH-Gal4*/+) and *relish* knockdown (*TH-Gal4* > *UAS-relish-RNAi*) flies was determined by confocal imaging. GFP-positive green signal depicts DA neuron clusters of the *Drosophila* brain. White boxes indicate DA neuron PPM3 cluster. Scale bars 100 μm. Enlarged representative views of the posterior DA clusters, protocerebral posterior medial (PPM3) and PPM2 are shown in the side panel. **(d)** Average number of dopaminergic neurons per cluster in controls (*TH-Gal4*/+) and *relish* knockdown (*TH-Gal4* > *UAS-relish-RNAi*) flies. DA neuron clusters: PPM2, PPM3 and protocerebral posterolateral (PPL1). Data are representative of at least three independent experiments. **P < 0.01; ***P < 0.001 based on two-way ANOVA with Tukey’s post-hoc test for multiple comparisons.

*Drosophila* innate immune signaling pathways are well-characterized and are highly conserved with vertebrate innate immune pathways^22,24^. Our studies show that NF-κB transcription factor, Relish is activated in response to PQ feeding without up-regulating the expression of AMPs downstream of the IMD pathway. In fact, the expression of the downstream AMPs was suppressed after PQ exposure. In order to determine the exact role of Relish in PQ-induced PD pathogenesis, we used UAS-RNAi transgenes under the control of *tyrosine hydroxylase* (*TH*)-*Gal4* to knockdown *relish* specifically in the DA neurons. As controls, we used both *TH-Gal4*>+ and *UAS-relish-RNAi* in the presence or absence of PQ in 2.5% sucrose. We observed that *relish* knockdown specifically in the DA neurons (*TH-Gal4* > *UAS-relish-RNAi)* leads to significantly increased survival (4 days in average) following PQ exposure compared to sucrose-fed control flies (p < 0.001) (Fig. 3a). To rule out the possibility that the knockdown of *relish* in the DA neurons could regulate food intake, we measured feeding rates using the blue food dye (1% FD&C Blue#1) mixed with either sucrose or PQ. These treatments did not lead to significant differences in the food intake abilities of the *relish* knockdown flies as compared to the corresponding controls (Fig. S4).

PQ exposure has been previously linked to altered movement phenotypes in *Drosophila* PD models^3,13^. To evaluate whether reduction of Relish levels in DA neurons could rescue the mobility defects induced by PQ, we employed a negative geotaxis assay, which is an indicator of the onset of PD pathogenesis-linked movement dysfunction. After ingestion of 5 mM PQ for 24 h, both the control groups (*TH-Gal4*>+ and *UAS-relish-RNAi*) exhibited resting tremors and bradykinesia, which are characteristic clinical symptoms associated with PD in human patients (Fig. 3b). We observed that *relish* knockdown in the DA neurons (*TH-Gal4* > *UAS-relish-RNAi)* significantly rescued flies against PQ-induced mobility defects as shown in Fig. 3b, consistent with improved survival during PQ exposure.

We have previously shown that PQ feeding causes the loss of specific clusters of DA neurons^3^. Since *relish* knockdown in DA neurons confers PQ resistance in terms of survival and mobility defects (Fig. 3a,b), we determined the relative contribution of Relish on PQ-induced loss of DA neurons in the *Drosophila* brain. The effect of 5 mM PQ exposure on DA neurons was detected after 48 h using GFP expression driven by *TH* promoter in *TH-Gal4* > *UAS-GFP* transgenic lines. The total number of DA neurons was significantly reduced in response to PQ ingestion in the WT flies (Fig. 3c). Consistent with earlier findings^3^, we also noticed that certain DA neuron clusters (protocerebral posterior medial: PPM2, PPM3, protocerebral posterolateral: PPL1) were more sensitive to PQ treatment (Fig. 3d). Targeted reduction of Relish protects against loss of DA neurons in response to PQ (Fig. 3c). Together, these data suggest that Relish activation is detrimental to survival following PQ exposure in PD pathogenesis.

### Paraquat-induced toxicity involves the IMD signaling pathway

**Figure 4:**
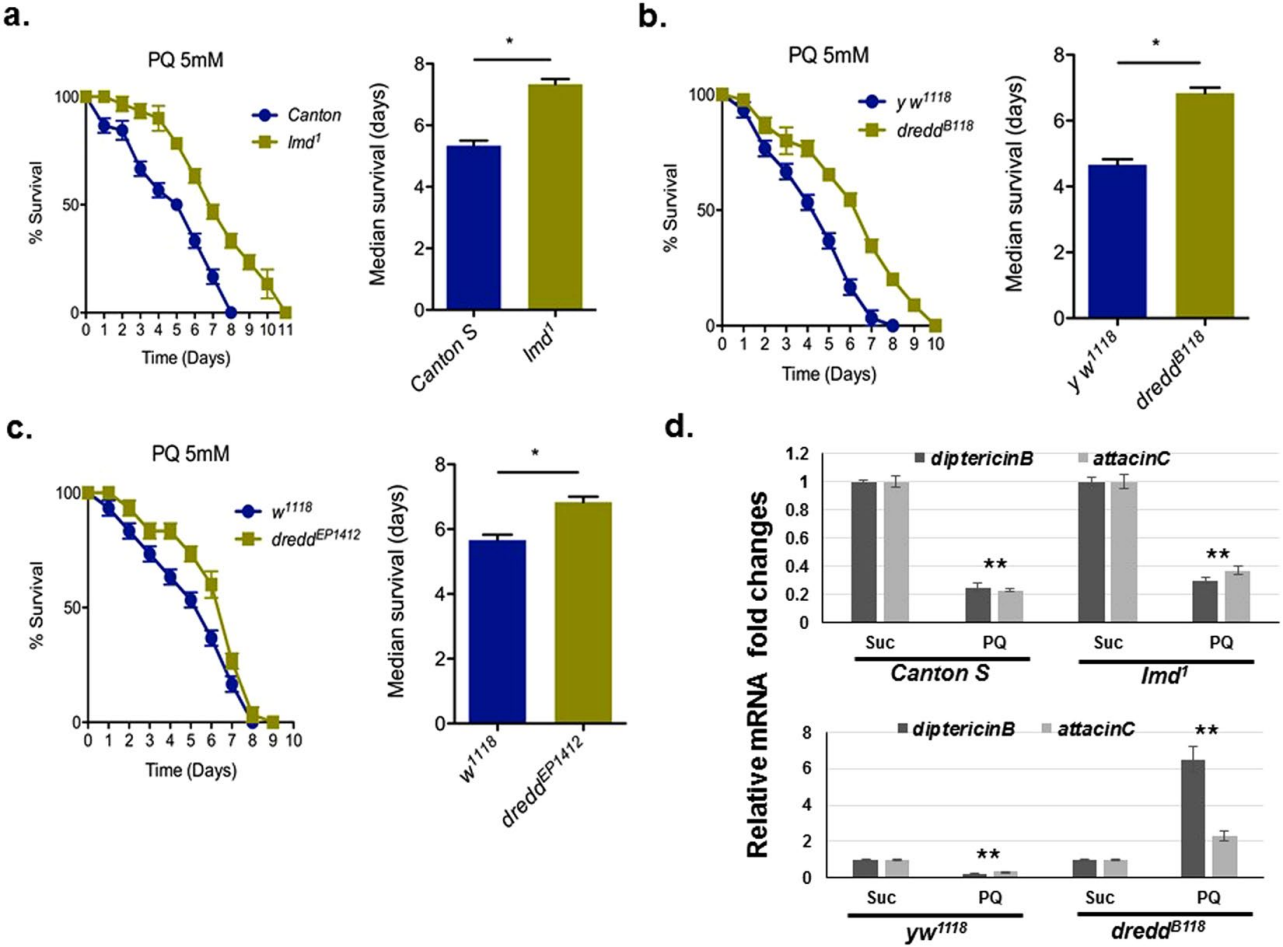
Paraquat toxicity is mediated through components of the IMD pathway. (**a)** Loss of *imd* improves survival upon PQ exposure. Survival assays were set up using *Imd-null* male flies along with the corresponding wild-type (*Canton S*) control following sucrose or 5 mM PQ treatment. Median survival (days) of flies are indicated. *P < 0.05 based on Mann-Whitney U test. Data are average of at least three independent experiments with 10–15 flies per group. **(b)** Improved survival in *Dredd* mutants in response PQ ingestion. Survival assays were set up using *Dredd* mutant (*Dredd*^*EP1412*^ and *Dredd*^*B118*^*)* male flies along with the corresponding wild-type controls following sucrose or 5 mM PQ treatment. Median survival (days) of flies are indicated. *P < 0.05 based on Mann-Whitney U test. Data are average of at least three independent experiments with 10–15 flies per group. **(c)** Effect of PQ exposure on AMP expression in *Imd* and *Dredd* mutant flies. RNA was isolated from the heads of wild type and mutant flies as indicated following 5 mM PQ or sucrose treatment for 12 h. Relative transcript levels of *diptericinB* and *attacinC* were analyzed using qRT-PCR and plotted after normalization with *rp49* levels as the internal control. Each data point represents the mean +/− SEM. mRNA fold changes are normalized to the sucrose-fed flies (assigned a value of 1). **P < 0.01 based on Mann Whitney’s *U*-test.

Our data indicate that Relish, which is the critical transcription factor downstream of the IMD signaling pathway, mediates PQ-induced PD pathogenesis in *Drosophila*. To determine whether additional components of the IMD pathway play a role in PQ-induced toxicity, we monitored the survival of *Imd* and *Dredd* mutant flies upon PQ ingestion. IMD is a death domain protein that mediates defense against Gram-negative bacterial infection through Relish-dependent induction of downstream AMPs and *Dredd* encodes a caspase downstream of IMD that mediates immune responses to Gram-negative bacteria and apoptotic pathways in *Drosophila*^60,61^. We observed significantly improved median survival of *Imd*^*1*^ (*Imd-null*) mutants in comparison to wild type *Canton S* controls following PQ treatment (Fig. 4a). To characterize *Dredd’s* role in mediating PQ-induced toxicity, we used two available mutant alleles: the hypomorphic *Dredd*^*EP1412*^ mutation and a *Dredd-null* (*Dredd*^*B118*^) mutant line that harbors a stop codon in the DREDD pro-domain^61^. Both mutants showed significantly improved median survival to PQ in comparison to their respective wild type controls, with slightly higher protection observed for *Dredd*^*B118*^ mutants (Fig. 4b,c). These data suggest that both IMD and the downstream caspase, DREDD are involved in mediating survival upon PQ exposure.

Because PQ treatment leads to suppression of AMP-coding genes downstream of the IMD pathway, we next evaluated the role of IMD and DREDD in PQ-mediated suppression of AMPs. Both the *Imd-null* and *Dredd-null* mutant flies along with their corresponding wild type flies were exposed to either sucrose alone or 5 mM PQ for 12 h, prior to RNA extraction from the heads followed by qRT-PCR using AMP-specific primers. As shown in Fig. 4d, the expression of both *diptericinB* and *attacinC* transcripts were suppressed in both the WT controls and *Imd-null* mutant flies in response to PQ, indicating that the upstream adaptor protein, IMD, is not required for PQ-mediated regulation of the AMPs. Surprisingly, these AMP genes were not suppressed in the *Dredd*^*B118*^ mutant flies in response to PQ, suggesting the involvement of the downstream caspase, DREDD in regulating PQ-mediated AMP expression.

### Paraquat-induced AMP regulation is mediated through Pirk

**Figure 5:**
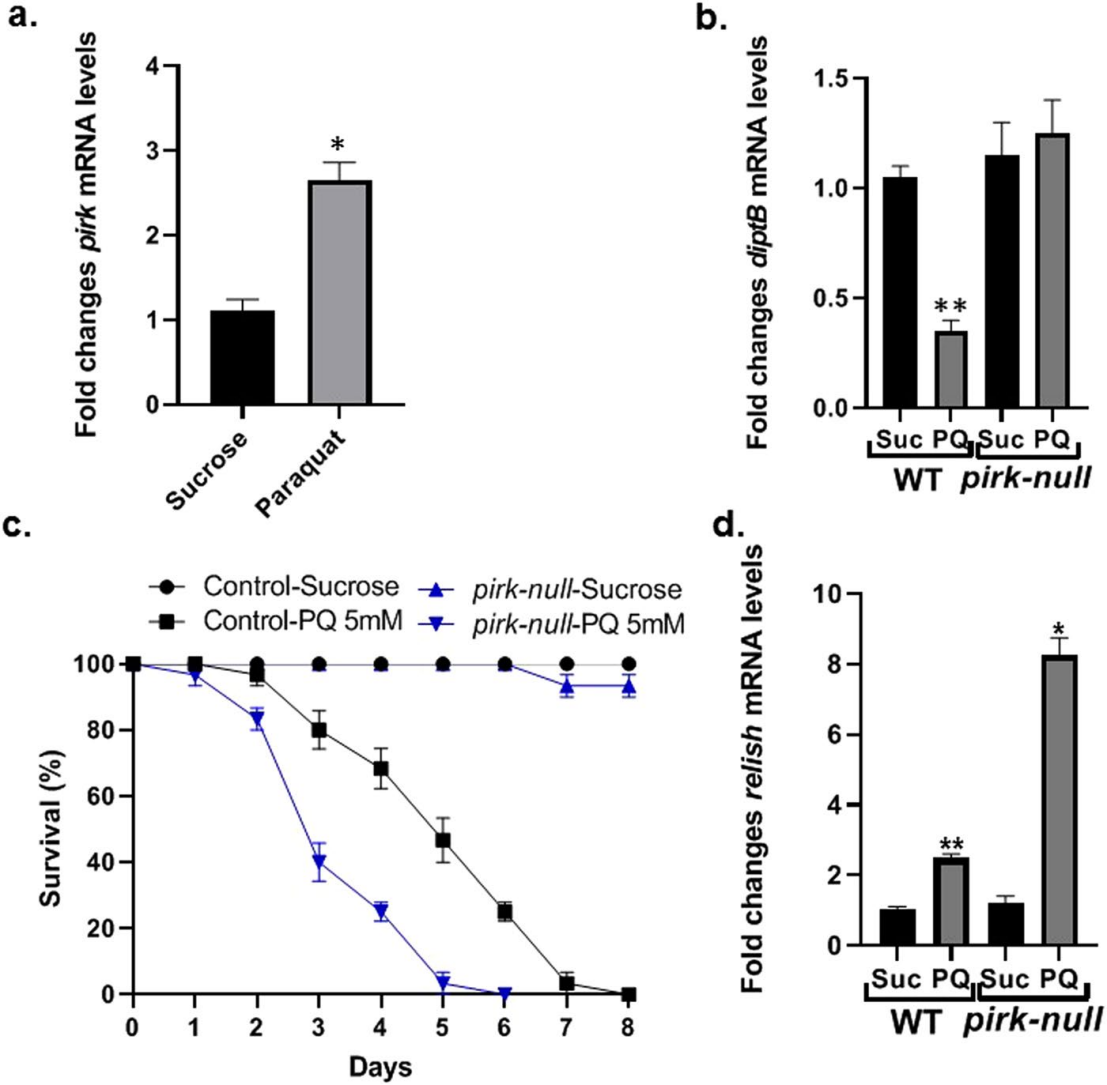
Pirk is required for paraquat-mediated AMP suppression. **(a)** PQ induces the expression of the negative regulator *pirk*. RNA was isolated from the heads of *Canton S* wild type flies following 5 mM PQ or sucrose treatment for 12 h. Relative transcript levels of *pirk* were analyzed using qRT-PCR and plotted after normalization with *rp49* levels as the internal control. Each data point represents the mean +/− SEM. mRNA fold changes are normalized to the sucrose-fed flies (assigned a value of 1). *P < 0.05 based on Mann Whitney U-test. **(b)** PQ-mediated AMP suppression requires Pirk. RNA was isolated from the heads of wild type *y w*^*1118*^ and *pirk-null* mutant flies following 5 mM PQ or sucrose treatment for 12 h. Relative transcript levels of *diptB* were analyzed using qRT-PCR and plotted after normalization with *rp49* levels as the internal control. Each data point represents the mean +/− SEM. mRNA fold changes are normalized to the sucrose-fed flies (assigned a value of 1) for each of the wild type and mutant strains. **P < 0.01 based on Mann-Whitney *U*-test. The results are representative of at least three independent experiments. **(c)** Loss of *pirk* increases PQ toxicity. Survival assays were set up using *pirk-null* male flies along with the corresponding wild-type (*y w*^*1118*^) control following sucrose or 5 mM PQ treatment. Log-rank test was used for survival analysis and statistically significant differences (*P* < 0.01) were observed between the *pirk-null* and the corresponding control group in response to PQ exposure. Data are average of at least five independent experiments with 10 flies per group. **(d)** Loss of *pirk* causes hyper-induction of *relish* transcript in response to PQ exposure. RNA was isolated from the heads of *pirk-null* mutant flies following 5 mM PQ or sucrose treatment for 12 h. Relative transcript levels of *relish* were analyzed using qRT-PCR and plotted after normalization with *rp49* levels as the internal control. Each data point represents the mean +/− SEM. mRNA fold changes are normalized to the sucrose-fed flies (assigned a value of 1). **P < 0.01 based on Mann Whitney U-test.

We have shown that PQ-mediated Relish activation does not lead to AMP induction downstream of the classical IMD pathway, as is typically the case following pathogen exposure^23^. Instead, we observed that AMP expression was suppressed more than 2-fold upon PQ feeding (Fig. 1c). Negative regulation plays an important role to ensure proper immune response against pathogens while preventing hyperactivation of the immune response that results in tissue damage and chronic inflammation^28^. We found that PQ exposure to 5 mM PQ for 12 h results in a 2.5-fold induction of *pirk*, which encodes a negative regulator of the IMD pathway that is itself regulated by Relish (Fig. 5a). Earlier studies have shown that Pirk overexpression suppresses the IMD pathway response following infection with Gram-negative bacteria^62^. To evaluate the role of Pirk in PQ-mediated suppression of AMPs, *pirk-null* mutant flies were exposed to 5 mM PQ for 12 h. As shown in Fig. 5b, *diptericinB* expression was reduced in the WT controls but not in *pirk-null* mutant flies in response to PQ. Our data indicate that *pirk* is required for suppression of AMP (*diptericinB)* expression in response to PQ treatment. To determine the effect of Pirk on PQ-mediated toxicity, we monitored the survival of *pirk*-*null* mutant flies upon PQ treatment. As shown in Fig. 5c, the survival rates of the control sucrose-fed WT and *pirk-null* mutant flies did not differ. However, *pirk-null* mutant flies had significantly (p < 0.01) lower survival rates compared with the WT control in response to PQ exposure, thereby indicating that loss of pirk increases PQ susceptibility. Since Pirk is a known negative regulator of the IMD pathway, we looked at the effect of Pirk mutation on *relish* expression upon PQ treatment. As shown in Fig. 5d, loss of *pirk* resulted in hyper-induction (more than 8-fold) of *relish* transcripts in response to PQ. Interestingly, *relish*-*null* mutant flies were not resistant to PQ toxicity in survival assays (Fig. S3). The higher sensitivity of *relish*-*null* mutant flies to PQ is likely due to the absence of functional *relish* during development which would affect multiple downstream genes affecting developmentally important cellular processes. These data suggest a novel role for Pirk with relevance to PQ-induced toxicity in *Drosophila* PD model.

### Paraquat exposure confers increased sensitivity to bacterial infection

**Figure 6:**
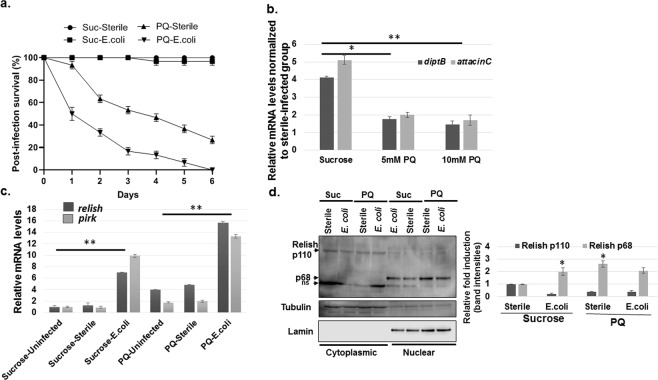
Paraquat exposure leads to increased susceptibility to Gram-negative bacterial infection. (**a)** *Canton S* wild type male flies were fed either 2.5% sucrose or 5 mM PQ for 12 h prior to *E*. *coli* infection. The pre-treated flies were then injected either with sterile needle or *E*. *coli* and survival was scored once every 24 h. Log-rank test was used for survival analysis and statistically significant differences (*P* < 0.001) were observed between the PQ-pretreated group in response to sterile or *E*. *coli* infection. Data are representative of at least three independent experiments with 10 flies per group. **(b)** PQ exposure suppresses AMP expression in response to Gram-negative bacterial infection. *Canton S* wild type male flies were fed either sucrose or 5 or 10 mM PQ for 12 h prior *E*. *coli* injection. Pre-treated flies were then injected either with sterile needle or *E*. *coli* followed by RNA extraction 6 h post-infection. Relative transcript levels of *diptB* and *attacin-C* were analyzed using qRT-PCR and plotted after normalization with *rp49* levels as the internal control. Each data point represents the mean +/− SEM. mRNA fold changes are normalized to the corresponding sterile-injected flies. *P < 0.05; **P < 0.01 based on Mann Whitney U-test. **(c)** The effect of PQ on *pirk* and *relish* transcripts in response to bacterial infection. *Canton S* wild type male flies were fed either sucrose or 5 mM PQ for 12 h prior to *E*. *coli* infection. The pre-treated flies were then either not infected, injected with sterile needle or *E*. *coli* followed by RNA extraction 6 h post-infection. Relative transcript levels of *relish* and *pirk* were analyzed using qRT-PCR and plotted after normalization with *rp49* levels as the internal control. Each data point represents the mean +/− SEM. mRNA fold changes are normalized to the sucrose-fed uninfected flies (assigned a value of 1). **P < 0.01 based on Mann Whitney U-test. **(d)** The effect of PQ on Relish localization in response to *E*. *coli* infection. *Canton S* wild type male flies were fed either sucrose or 5 mM PQ for 12 h prior to *E*. *coli* infection. The pre-treated flies were then either injected with sterile needle or infected with *E*. *coli* followed by nuclear fractionation and immunoblotting using the specified antibodies. The full-length Relish p110 and p68 are indicated by arrows. The same blots were probed with anti-Lamin and anti-Tubulin as the nuclear and cytoplasmic markers for the fractionation protocol. The band intensities were quantitated using the LI-COR C-DiGit Blot scanner software and plotted after normalization with Lamin and Tubulin. *P < 0.05 based on Student’s *t* test.

*Drosophila* mounts an robust immune response to combat and destroy bacterial infection by inducing AMP expression^22,25^. The failure to induce AMPs to efficiently clear bacterial infections results in higher mortality rates^25^. Based on our data showing PQ-mediated suppression of AMPs, we hypothesized that PQ exposure would increase susceptibility to Gram-negative bacterial infection. To test this hypothesis, we poked into the thorax of *Canton S* WT flies with a concentrated culture of the Gram-negative bacteria *Escherichia coli* (*E*. *coli*) to activate the innate immune response and then monitored flies’ survival. The *Canton S* WT flies were fed either sucrose or 5 mM PQ containing sucrose for 12 h prior to *E*. *coli* infection and the number of flies was scored daily for survival. In addition, sterile injections performed on sucrose and PQ-fed flies show that inflammation resulting from the needle injury alone is not sufficient to kill the flies. The results shown in Fig. 6a demonstrate that PQ pre-treatment leads to a significantly increased sensitivity to *E*. *coli* infection as compared to the sucrose-fed flies. Since inducible AMPs are important for the immune defense in *Drosophila*, we next investigated whether the increased susceptibility could be due to the reduced induction of AMPs following infection in PQ treated flies. Strikingly, PQ pre-treatment abrogated AMP induction (*diptericinB and attacinC*) in a dose-dependent manner following *E*. *coli* infection as compared to the sucrose-fed control flies (Fig. 6b). The data suggest that PQ-mediated suppression of AMPs leads to a compromised immune response against bacterial infection. The higher mortality in the PQ-fed flies is likely due to their inability to clear the infection effectively. Since Relish is required for AMP expression downstream of the Imd pathway, we sought to detect the effect of PQ exposure on the *relish* transcript levels post-infection. In addition, we examined the levels of *pirk* in the sucrose and PQ fed flies post-infection as Pirk is a known downstream target of Relish in the IMD pathway. As depicted in Fig. 6c, the transcript levels of *relish* and *pirk* were induced in response to *E*. *coli* infection in both the sucrose and PQ-fed flies. However, *relish* induction does not translate to the activation of AMP expression downstream of the IMD pathway. We further explored the localization of the active Relish p68 in the sucrose and PQ-fed flies in response to *E*. *coli* infection. As shown in Fig. 6d, the active nuclear Relish p68 is induced in the sucrose-fed flies in response to *E*. *coli* infection (compare sucrose-fed sterile vs *E*. *coli*). PQ treatment alone causes nuclear translocation of the active Relish p68 and a gradual decrease in the full-length cytoplasmic Relish p110 form (Fig. 6d). However, upon PQ pre-treatment, the nuclear Relish p68 fails to initiate AMP gene transcription following *E*. *coli* infection. Taken together, the data suggest that PQ exposure increases susceptibility to Gram-negative bacterial infection due to the inability of active Relish to induce AMP expression downstream of the IMD pathway, thereby leading to a compromised immune response and more rapid death of infected flies.

## Discussion

Neuroinflammation is associated with several neurodegenerative diseases, including AD, PD, and multiple sclerosis (MS), that contribute to neuronal dysfunction and death^5^. Populations of activated microglia have been observed in areas of neuronal damage and degeneration in post-mortem brains of patients suffering from PD and AD, and subsequent research has shown that inflammatory responses are functionally linked to disease progression^6–8^. The involvement of dysregulated inflammation impedes deployment of therapeutic intervention capable of slowing or blocking disease progression. A serious confounding factor is that most neurodegenerative diseases are not caused by highly penetrant, monogenic variants, but rather, appear to be rooted in combinations of genetic background and exposure to generally ill-defined environmental factors. As the vast majority of PD cases are sporadic, the underlying mechanisms of disease progression remain poorly understood.

In this study, we employed transcriptomic profiling in response to transient PQ exposure in *Drosophila* to identify pre-symptomatic gene signatures involved in the progression of PD, prior to the manifestation of movement dysfunction. The inflammatory responses to neurodegeneration in the adult *Drosophila* brain mirror quite strongly the responses to neurodegeneration in mammalian models and in human disease^63^. Our RNAseq data demonstrate that PQ exposure triggers transcriptional changes in genes involved in innate immune response pathways in *Drosophila*. We show that PQ treatment activates genes associated with *Drosophila* hemocytes, which are macrophage-like cells involved in phagocytosis and inflammation. This finding is in agreement with our previous data showing neuroinflammatory responses through the induction of NOS in response to PQ treatment^33^. Moreover, involvement of hemocytes has also been implicated in the progression of AD in the fly model^64^. Interestingly, we observed that other innate immune genes, particularly those encoding AMPs, downstream of both the Toll and IMD pathways were suppressed in response to PQ exposure. Contrary to our findings, earlier reports suggest a link between increased expression of AMPs and neurodegeneration^27,29,30^. One possible explanation for this difference could be that we have employed a pre-symptomatic disease onset model, as opposed to other reported models that mainly focus on degeneration stage processes. A pre-symptomatic strategy can both optimize target manipulation by genetic or drug intervention and highlight key biomarkers with potential for early diagnosis and treatment. Our results indicate that PQ-mediated suppression of AMP expression might impair the host innate immune system leading to decreased ability to fight bacterial infection. In this study, we focused on AMPs downstream of the IMD pathway due to the involvement of Relish in PQ-mediated neurodegeneration of DA neurons. Further dissection of PQ-mediated regulation of the Toll pathway may aid our understanding of the mechanistic link between aberrant innate immune response and PD pathogenesis.

In addition to genes involved in the innate immune response, the transcriptomic analysis identified genes, whose mammalian orthologs are also implicated in PD pathogenesis. For example, we observed increased expression of *Gadd45* transcript in fly heads following PQ exposure, while DA neurons-specific knockdown of this gene using RNAi led to increased PQ sensitivity, suggesting a neuroprotective effect of GADD45 against PQ-induced neurotoxicity in *Drosophila*. In alignment with our findings, GADD45α has been shown to be protective against MPP+ toxicity in human dopamine neuroblastoma cells^42^. Furthermore, our results revealed induction of chaperone responses through the upregulation of Hsps (Hsp22, Hsp26, Hsp68) upon PQ exposure. Hsps have been shown to play neuroprotective roles in both mammalian and *Drosophila* models of neurodegenerative disease, including PD^44^. Interestingly, earlier studies show neuron-specific expression of Hsp26 and Hsp70 improves life span and enhances stress resistance in PQ and α-synuclein-induced cellular toxicity in both *Drosophila* and mammalian models^46,47^. Noticeably, we also identified genes involved in the glutathione metabolic responses (*GstD2*, *GstD5*, *GstE7*, *GstE8*) as PQ-responding genes. The neuroprotective role of GSTs has been established in several models of PD, including *Drosophila* where overexpression of GST has been shown to ameliorate neurodegeneration^48,49^. These findings justify the use of our toxin-induced neurodegeneration model system in *Drosophila* to successfully identify potential therapeutic targets in PD. However, further studies are warranted with different PQ doses and duration to build a temporal landscape of pre-symptomatic gene expression signatures to discover novel molecular targets relevant to prediction of future disease and potential therapeutic targets.

Our data indicate that the NF-κB transcription factor Relish is required for PQ-induced neurodegeneration and suggest a role in environmental toxin-induced PD pathogenesis. Relish regulates the inducible innate immune response in *Drosophila* through signal-dependent translocation into the nucleus resulting in increased expression of AMPs downstream of the IMD pathway^24,25^. Activation of Relish in glial cells has been shown to be responsible for neuron loss in a model of ataxia–telangiectasia, while neuron-specific overexpression of Relish-dependent AMP genes is sufficient to cause neurodegeneration^29–31,65^. Here, we demonstrate that PQ treatment mediates nuclear translocation of active Relish. In addition, we show that targeted knockdown of *relish* specifically in the dopaminergic neurons significantly improves survival, climbing performance and rescues DA neuron loss following PQ exposure. Our results suggest that activation of Relish contributes to environmental toxin-induced neurodegeneration. Consistent with our findings, targeting NF-κB activation has been shown to be an effective therapy against neurodegeneration in murine PD models^66^. It would be interesting to investigate the role of Relish in different cell types including glial cells and hemocytes in response to PQ-induced neurodegeneration.

Oxidative stress plays a major role in the loss of dopaminergic neurons due to excessive production of ROS^67^. Noticeably, the RNAseq screen revealed upregulation of genes involved in redox reactions upon PQ exposure (Table 1). In mammalian PD models, JNK signaling pathway plays an important role in regulating the cellular processes such as oxidative stress and apoptosis^14^. Our data show that JNK is activated by phosphorylation following PQ treatment, while pharmacological inhibition of JNK increases survival in WT flies. In accordance with our data, knockdown of the *basket* gene in the dopaminergic neurons has been shown to improve survival and climbing ability in *Drosophila*^68^. Prolonged JNK activation has been implicated in exacerbating disease phenotypes in both AD and PD models^69,70^. In contrast, JNK has also been shown to be protective in diverse PD models because JNK can be both pro- and anti-apoptotic depending on cell type, nature and duration of the activation stimulus, interaction with other signaling pathways and genetic backgrounds^13,14,58,71^. Consistent with our observations, another group demonstrated that inhibition of JNK activity using SP600125 confers protection against PQ toxicity^59^. Furthermore, increased JNK signaling has been implicated in PQ-induced neurodegeneration and active JNK has been reported in *Drosophila parkin* mutants^14,72^.

We demonstrate that PQ exposure induces nuclear translocation of Relish p68 (Fig. 2a). Interestingly, activated Relish fails to induce the expression of downstream target AMPs genes upon PQ treatment, thus compromising survival following infection with Gram-negative bacteria. Our data suggest that PQ-induced toxicity is mediated by components of the IMD pathway, including the adaptor protein, IMD, and the downstream caspase, DREDD^23^. Interestingly, both the *Imd* and *Dredd* mutant flies show increased resistance to PQ toxicity in survival assays. DREDD is required for the activation and nuclear translocation of active Relish in response to bacterial infection. In addition to its role in antibacterial response, the caspase DREDD is involved in regulating apoptotic pathways in *Drosophila*^60^. Interestingly, caspase-8 activation has been reported in both PD patients as well as environmental-toxin induced PD model in mice^73^. In a fly model of retinal degeneration, DREDD but not IMD plays a role in photoreceptor degeneration^74^. Our work shows that both of these factors mediate survival to PQ-induced toxicity and future studies will determine their involvement in PQ-mediated DA neuronal degeneration.

**Figure 7:**
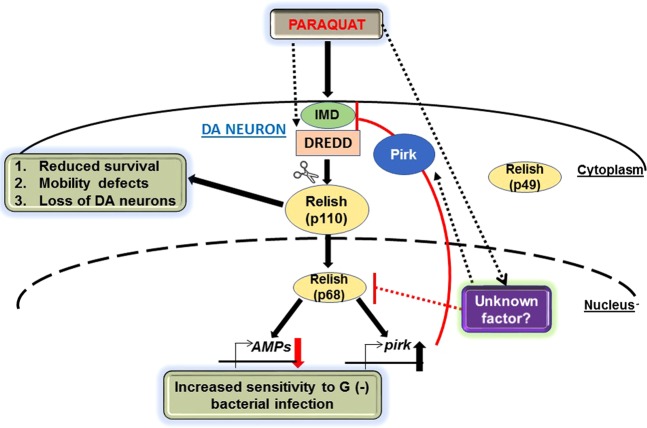
Schematic representation of paraquat-mediated innate immune responses in a *Drosophila* PD model. Short-term PQ exposure regulates genes involved in the *Drosophila* innate immunity. PQ activates full-length Relish p110 by proteolytic cleavage and induces nuclear translocation of Relish p68. In the dopaminergic neurons, PQ-induced Relish activation leads to poor survival, mobility defects and loss of specific dopaminergic neuron clusters. PQ-mediated toxicity involves components of the IMD pathway, the adapter protein, IMD and the downstream caspase, DREDD. PQ inhibits Relish-dependent induction of AMPs through activation of pirk, a negative regulator of the IMD pathway. PQ-mediated suppression of AMPs disrupts the innate immune response thereby increasing susceptibility to Gram-negative bacterial infections. On the other hand, PQ might also directly activate other unknown factors which might interact with either Relish, DREDD and/or Pirk and lead to suppression of AMPs. Dotted lines represent hypothetical pathways regulating immune responses downstream of PQ.

NF-kB signaling is tightly regulated by negative regulators to avoid prolonged immune responses that are detrimental to the host^28^. Our results show that PQ induces the expression of *pirk*, a gene encoding a negative regulator of the IMD pathway. In addition, we show that Pirk is required for PQ-mediated suppression of AMPs suggesting a novel function of Pirk in environmental toxin-induced PD pathogenesis. Elevated levels of the negative regulator Pirk might activate or interact with another unknown factor to prevent nuclear active Relish p68 from inducing downstream AMP targets. In the context of bacterial infection, *pirk* induction depends on Relish and Pirk regulates IMD signaling at the level of the adaptor protein IMD, which is downstream of the receptor PGRP-LC^25,62^. Our data suggest that DREDD but not the upstream adaptor protein, IMD is required to regulate AMP expression in response to PQ. Therefore, it is possible that PQ-mediated activation of Relish and AMP expression might be regulated through an alternative noncanonical pathway as observed in *ATM* mutants^30^. A recent study demonstrated that Relish becomes activated in response to infection with the invertebrate iridescent virus 6 (IIV6) and that its translocation to the nucleus results in suppression of AMPs gene expression^75^. The failure of active Relish to induce downstream AMP gene expression upon PQ exposure suggests that transcriptional inhibition could occur at the promoter-binding site. Our results show that loss of *pirk* results in increased PQ sensitivity leading to higher mortality due to induction of *relish* levels. We speculate that PQ exposure might directly activate DREDD or additional factors responsible for nuclear translocation of Relish resulting in *pirk* induction and subsequent inhibition of AMPs as depicted in Fig. 7. Therefore, future studies will focus on elucidating the mechanisms regulating Relish activation upon PQ exposure. In addition, further studies are required to understand the precise role of Pirk in the regulation of innate immune responses following PQ treatment. Since our data show that PQ downregulates AMPs and that loss of *relish* in the DA neurons improves the PQ-induced parkinsonian phenotypes, further studies are warranted to dissect the exact role of AMPs in PQ-mediated toxicity.

There is a growing body of evidence suggesting higher susceptibility to infection in PD patients and in PD disease models, although the precise mechanism remains to be elucidated. Higher infection burden has been associated with PD pathogenesis due to increased levels of serum inflammatory cytokines and α-synuclein^76,77^. Additionally, high prevalence of small intestinal bacterial overgrowth and higher incidence of respiratory tract and urinary tract infections has been reported in PD patients^78,79^. Moreover, PD patients exhibit higher susceptibility to infections affecting the central nervous system and sepsis^80^. Interestingly, Parkin, an E3 ubiquitin ligase associated with early onset PD also modulates innate immune responses; loss of parkin leads to a defective immune response against bacterial infection in flies and murine models^81^. Genome-wide association studies link leucine rich repeat kinase 2 (LRRK2) variants, related to one of the leading heritable forms of PD, to higher susceptibility to the autoimmune Crohn’s disease and *Mycobacterium leprae* infection^82,83^, reinforcing a strong link between elevated risk of infection and PD. Consistent with these findings, we demonstrate that in *Drosophila*, PQ treatment results in an increased susceptibility to Gram-negative bacterial infection due to suppressed AMP expression. Healthy flies normally survive infection with a non-pathogenic strain of *E*. *coli* as observed in the sucrose-fed group^84^, whereas flies exposed to the environmental toxin PQ exhibit compromised immune response and higher mortality. Moreover, increased bacterial susceptibility has also been reported in another genetic PD model using *parkin* deficient flies^81^. Therefore, our observation of suppressed AMP expression as a response to PQ exposure suggests a possible mechanism for the increased risk of infections in PD patients.

Our study provides strong evidence for the involvement of the innate immune response in environmental toxin-induced PD pathogenesis. We also show that PQ-mediated dysregulation of the innate immune response is associated with increased susceptibility to *E*.*coli* infection. Further studies of pre-symptomatic immune systems are warranted for new therapies aimed at modulating the immune system’s response during PD, thereby leading to improved clinical outcomes for disease.

## Materials and Methods

### Drosophila strains and culture maintenance

Stocks were raised and maintained at 25 °C on standard medium (Bloomington Stock Center Recipe) containing cornmeal, corn syrup, yeast, and agar. The control genotype for all experiments was *Canton S* or the appropriate heterozygous control, and flies used for all adult assays were three to five-day old males unless otherwise noted. The following stocks were purchased from the Bloomington *Drosophila* Stock Center at Indiana University: *Canton S*, *y w*^1118^, *w*^1118^, *UAS-2XEGFP*, *pirk*^*EY00723*^, *w*^1*118*^*; Rel*^*E20*^, *UAS-GFP*, *UAS-Rel*^*RNAi#33661*^, and *UAS-Rel*^*RNAi#28943*^, *Imd*^*1*^, *Dredd*^*B118*^, *Dredd*^*EP1412*^. *TH-GAL4* was a gift from J. Hirsh (University of Virginia, Charlottesville, VA).

### RNAseq analysis

Transcriptomic profiling was performed using four independent biological replicates per feeding. Age-matched adult *Canton S* male flies were fed either 2.5% sucrose (control) or 5 mM PQ in 2.5% sucrose for 12 h and the heads were collected for RNA extraction. Total RNA was extracted using TRIzol Reagent (Thermo Fisher Scientific) from the fly heads following sucrose or PQ ingestion (thirty males/group). The concentration and integrity of the extracted total RNA were estimated using the Qubit® 2.0 Fluorometer (Invitrogen) and Agilent 2100 Bioanalyzer (Applied Biosystems, Carlsbad, CA, USA), respectively. For paired-end library preparation, NEBNext mRNA Library Prep Reagent Set for Illumina was used and the library concentration was analyzed by utilizing a DNA 1000 Chip on an Agilent 2100 Bioanalyzer. Paired-end sequencing (25 million, 50-bp, paired-end reads) was performed using a 200 Cycle TruSeq SBS HS v4 Kit on an Illumina HiSeq2500 sequencer (Illumina, Inc., San Diego, CA, USA) at the HudsonAlpha Institute for Biotechnology, Huntsville, AL. TopHat v2.0 were used to map raw reads to the reference Drosophila melanogaster genome dm^3^ (Fig. S1). The transcript abundance was quantified for the final read list using Trimmed Means of M-values as the normalization method. The p-value of the differentially expressed gene list were estimated by z-score calculations using Benjamini Hochberg corrections of 0.05 for false-discovery rate. Differential gene expression was calculated on the basis of fold changes between the control sucrose and PQ-fed groups. A gene ontology (GO) analysis for enriched biological process was performed on the list of differentially expressed mRNAs between samples using DAVID v6.8 and the GO data represented in Table 1 were filtered with thresholds that only show enriched biological processes categories with 3 or more associated genes.

### Paraquat treatment, JNK inhibitor and survival assay

Ten males of specified genotype, aged 3–5 days post-eclosion, were fed daily on filter paper saturated with specified concentrations of paraquat (Methyl viologen dichloride hydrate, Sigma-Aldrich) in 2.5% sucrose (Sigma-Aldrich) or with 2.5% sucrose only. Mortality was monitored daily for ten days. PQ treatment was performed with at least five independent biological replicates of 10 males each for each genotype tested. The JNK inhibitor, SP600125 was purchased from Sigma-Aldrich.

### Climbing assay

The mobility of adult male flies from each treatment group was recorded using a negative geotaxis climbing assay. Ten flies per treatment group were placed in an empty plastic vial and gently tapped to the bottom. The percentage of flies that crossed a line 5 cm from the bottom of the vial in 20 sec were calculated. Each biological replicate was assayed three times at 1 min intervals and the percentages averaged. Statistical significance between different genotypes was calculated using One-way analysis of variance (ANOVA) for *P* < 0.05.

### Confocal microscopy

Brains from adult flies were processed for confocal imaging as described earlier^3^. Briefly, brains were dissected in 1x phosphate buffered saline with Tween20 (PBST), fixed in 4% paraformaldehyde for 20 min in a 24 well plate, and washed three times using 1x PBST. GFP-positive dopaminergic neurons were detected using a chicken polyclonal anti-GFP antibody (Abcam) and Alexa fluor® 488 goat anti-chicken secondary antibody (Thermo Fisher Scientific). Brains were mounted on slides using ProLong Gold Antifade Mountant with DAPI (Thermo Fisher Scientific). Whole mounts of dissected brains were imaged using a Nikon C2 DUVb confocal laser scanning microscope. The GFP-positive cells of specific DA clusters were counted based on confocal Z-stacks of whole mount brains.

### Western blot analysis

25–30 adult fly heads were homogenized in RIPA buffer (Amresco) supplemented with 2 mM DTT and 1x protease inhibitor cocktail (Amresco). The homogenates were centrifuged at 12,000 × g for 5 min at 4 °C. Equal amounts of samples were mixed with 4X laemmli sample buffer (LDS) and heated at 70 °C for 10 min, briefly centrifuged, and subjected to SDS-PAGE using 4–12% NuPage Bis-Tris minigel and MOPS SDS Running buffer. Proteins were transferred to nitrocellulose or PVDF membranes (Bio-Rad) using iBlot Transfer Stacks, blocked in 2.5% dry nonfat milk and immunoblotted using one of the following antibodies: anti-Relish (RayBiotech Inc) (1:1000), anti-Tubulin (Sigma-Aldrich) (1:2500), anti-Lamin (Development Studies Hybridoma Bank) (1:2000), anti-JNK (Cell Signaling) (1:1000), anti-phosphoJNK (Cell Signaling) (1:1000). The appropriate anti-mouse or anti-rabbit horseradish peroxidase-conjugated secondary antibody was used, and signal was detected using Supersignal West Pico Chemiluminescent Substrate (Thermo Fisher Scientific) and LI-COR C-DiGit Blot scanner.

### Cell fractionation

Approximately 50 adult fly heads were collected per experimental group and nuclear fractionation was performed using NE-PER Nuclear and Cytoplasmic Extraction Kit (Thermo Fisher Scientific) according to the manufacturer’s protocol. Briefly, the heads were homogenized in 100 μL Cytoplasmic Extraction Reagent I, containing protease inhibitors, and samples were vortexed and allowed to incubate on ice for 10 min, followed by the addition of 5.5 μL of Cytoplasmic Extraction Reagent II and centrifugation at 10,000 rpm for 5 min at 4 °C. The supernatant was saved at −80 °C as the cytoplasmic fraction. The pellet was resuspended in 50 μL of Nuclear Extraction Reagent I (1X Protease Inhibitor, Thermo Scientific, IL USA). The samples were incubated on ice for 40 mins followed by centrifugation at 10,000 rpm for 10 mins at 4 °C. The resulting supernatant (nuclear fraction) was collected and stored at −80 °C for Western blot analysis.

### Quantitative Real time RT-PCR

Total RNA was extracted from 30 adult fly heads using TRIzol Reagent (Thermo Fisher Scientific), and cDNA was prepared from 0.5–1 μg total RNA using the High Capacity cDNA Reverse Transcription kit (Applied Biosystems). Triplicate cDNA samples were amplified using the iQ SYBR Green Supermix (Biorad) in a StepOnePlus Real time PCR System according to the manufacturer’s protocols. The relative levels of specified transcripts were calculated using the ΔΔCt method, and the results were normalized based on the expression of *ribosomal protein L32* (*RpL32/RP49*) as the endogenous control within the same experimental setting.

### Bacterial infection

*Canton S* adult flies were infected by poking the thorax with a fine needle dipped into a concentrated overnight grown culture of *Escherichia coli* (ATCC#11775 strain). For gene expression analysis, triplicates of male flies were snap frozen in liquid nitrogen 6 h post-infection. Frozen heads were homogenized in TRIzol reagent (Invitrogen Life Technologies), and the total RNAs extracted according to the manufacturer’s instructions. For survival assays, 30 flies were infected per experimental group as described above and their survival was scored daily at 25 °C.

### Statistical analysis

Data were analyzed using GraphPad Prism 8 software (GraphPad Software, Inc., La Jolla, CA). Statistical significances of gene expression were determined using a non-parametric Mann Whitney U-test. Statistically significant differences between survival curves were determined by log-rank test. Student’s *t* test was used as a parametric test for comparing differences between two groups. Results are expressed as mean ± SEM. *p* < 0.05 was considered statistically significant. Details of analysis are specified in the figure legends.

## Supplementary information

Supplementary Info

Supplementary Table

## Data Availability

All the data generated or analyzed during this study are included in this published article or available from the corresponding author upon request. RNAseq expression data are available in the Gene Expression Omnibus (GEO) repository through Accession number: GSE135919.
