# Supplementary material for: Innate immune responses to paraquat exposure in a Drosophila model of Parkinson’s disease
Source: Sci Rep. 2019 Sep 3;9:12714. doi: 10.1038/s41598-019-48977-6 (PMC6722124; doi:10.1038/s41598-019-48977-6)
Supplement: Supplementary file 1 — Supplementary Info [file 41598_2019_48977_MOESM1_ESM.docx]

**Innate immune responses to paraquat exposure in a *Drosophila* model of Parkinson’s disease**

Urmila Maitra*, Michael N. Scaglione, Stanislava Chtarbanova and Janis M. O’Donnell

Department of Biological Sciences, University of Alabama, Tuscaloosa, Alabama 35487-0344

*Corresponding author:

Dr. Urmila Maitra

Department of Biological Sciences, Box 870344,

University of Alabama,

Tuscaloosa, AL 35487-0344.

Email: [umaitra@ua.edu](mailto:umaitra@ua.edu)

**Supplementary Information**

| **SL#** | **GSL_ID** | **# Reads** | **PE Reads** | **PF Reads** | **PF PE Reads** |
| --- | --- | --- | --- | --- | --- |
|  |  |  |  |  |  |
| SL106349 | 3124-JO-0001 | 45,893,398 | 22,946,699 | 42,434,083 | 21,217,041 |
| SL106350 | 3124-JO-0002 | 45,496,220 | 22,748,110 | 42,256,455 | 21,128,227 |
| SL106352 | 3124-JO-0004 | 52,537,302 | 26,268,651 | 48,832,348 | 24,416,174 |
| SL106354 | 3124-JO-0007 | 50,777,358 | 25,388,679 | 47,687,683 | 23,843,842 |
| SL106355 | 3124-JO-0008 | 48,847,450 | 24,423,725 | 45,678,275 | 22,839,138 |
| SL106357 | 3124-JO-0010 | 59,267,770 | 29,633,885 | 55,205,005 | 27,602,502 |
| SL106358 | 3124-JO-0011 | 57,641,480 | 28,820,740 | 53,563,373 | 26,781,687 |
| SL106360 | 3124-JO-0013 | 52,559,332 | 26,279,666 | 49,137,702 | 24,568,851 |
|  |  |  |  |  |  |

**Fig. S1** **RNAseq mapped reads.**

Transcriptomic profiling was performed using *Canton S* male flies exposed to either sucrose or paraquat. Paired-end sequencing (25 million, 50-bp, paired-end reads) was performed using a 200 Cycle TruSeq SBS HS v4 Kit on an Illumina HiSeq2500 sequencer (Illumina, Inc., San Diego, CA, USA) at the HudsonAlpha Institute for Biotechnology, Huntsville, AL. TopHat v2.0 were used to map raw reads to the reference *Drosophila melanogaster* genome dm3.

Sample ID:

2.5 % Sucrose: 3124-JO-0001; 3124-JO-0004; 3124-JO-0007; 3124-JO-0010

5mM PQ: 3124-JO-0002; 3124-JO-0008; 3124-JO-0011; 3124-JO-0013

**Supplementary Table S1 (.xlsx file attached)**

Description of differentially expressed genes in 2.5% Sucrose and 5 mM PQ-fed flies.

Fold change (FC) gene expression profile ([PQ] vs [Control 2.5 % Sucrose]).

**
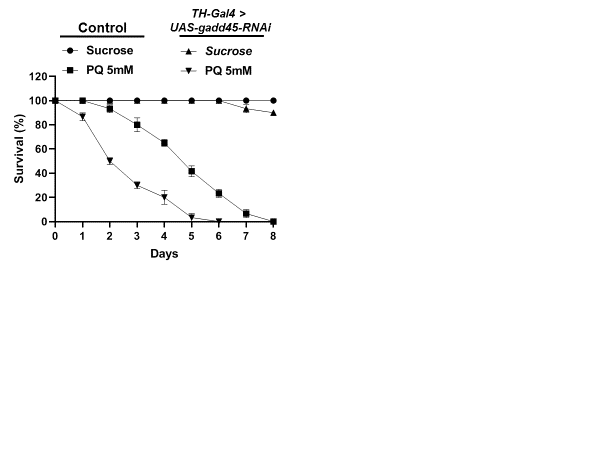
**

**Fig. S2 Knockdown of *gadd45* in the DA neurons increases PQ sensitivity.**

Survival assays were set up using male flies with *gadd45* knockdown in the DA neurons (*TH-Gal4>UAS-gadd45-RNAi*) and the corresponding control (*TH-Gal4/+*) following 2.5% sucrose or PQ (5 mM) treatment. Log-rank test was used for survival analysis and statistically significant differences (p<0.001) were observed between the *gadd45* knockdown (*TH-Gal4>UAS-gadd45-RNAi)* and the corresponding control groups (*TH-Gal4>+*) in response to PQ exposure. Data are representative of five independent experiments with 10 male flies per treatment group.


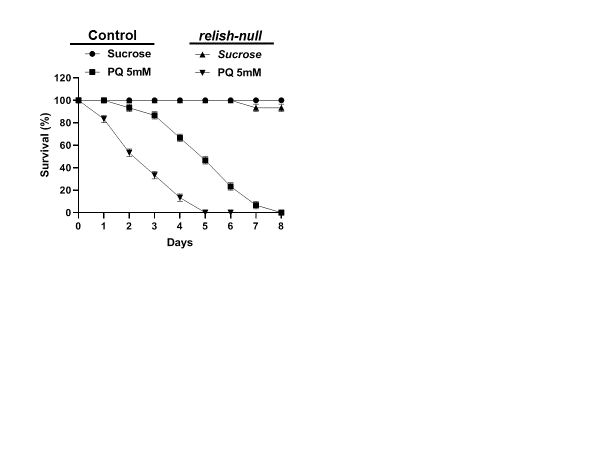


**Fig. S3 *relish-null* flies display increased PQ sensitivity.**

Survival assays were set up using *relish-null* male flies with and the corresponding control (*w^1118^*) following 2.5% sucrose or PQ (5 mM) treatment. Log-rank test was used for survival analysis and statistically significant differences (p<0.001) were observed between the *relish-null* and the corresponding control group in response to PQ exposure. Data are representative of ten independent experiments with 10 male flies per treatment group.


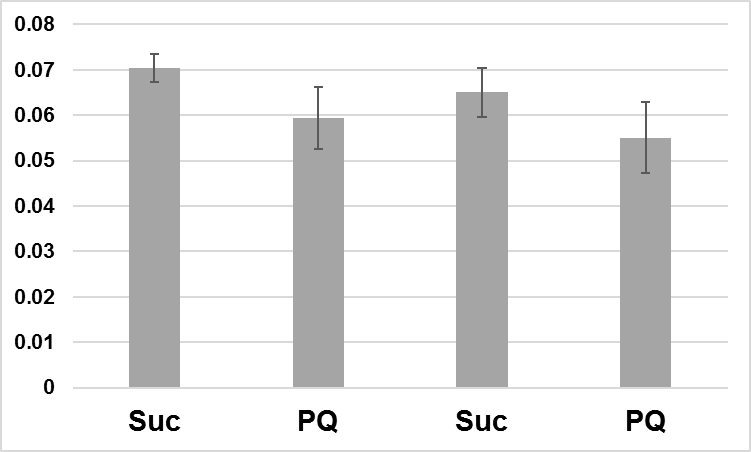


**Food uptake (absorbance)**

ns (p>0.05)

ns (p>0.05)

**WT Control**

***TH-Gal4>relish-RNAi***

**Fig. S4 Knockdown of *relish* in the DA neurons does not affect food intake ability.**

Both the WT control *(TH-Gal4/+)* and the *TH-Gal4>relish-RNAi* flies were fed either 2.5% sucrose or PQ (5 mM) containing the blue food dye (1% FD&C Blue#1) for 12 h to quantitate food intake. The flies were washed and homogenized in 1X PBS containing 1% Triton X-100 followed by centrifugation at 12,000 rpm for 5 min and the supernatants were measured at OD630 nm. Data were analyzed using one-way ANOVA and error bars indicate the standard deviation. No statistically significant differences (p>0.05; ns=non-significant) in the food intake abilities were observed between the *TH-Gal4>relish-RNAi* flies and the corresponding control group in response to both Sucrose and PQ feeding. Data are representative of five independent experiments with 10 male flies per treatment group.
